# Biosynthesis and characterization of zinc oxide nanoparticles using *Nigella sativa* against coccidiosis in commercial poultry

**DOI:** 10.1038/s41598-023-33416-4

**Published:** 2023-04-21

**Authors:** Najam-ul Lail, Adeel Sattar, Muhammad Ovais Omer, Mian Abdul Hafeez, Abdur Rauf Khalid, Sammina Mahmood, Muhammad Abubakr Shabbir, Waqas Ahmed, Muhammad Tahir Aleem, Abdulaziz Alouffi, Mashal M. Almutairi

**Affiliations:** 1 Department of Pharmacology and Toxicology, Faculty of Bio-Sciences, University of Veterinary and Animal Sciences, Lahore, Pakistan; 2 Department of Parasitology, Faculty of Veterinary Sciences, University of Veterinary and Animal Sciences, Lahore, Pakistan; 3 Deparment of Livestock and Poultry Production, Faculty of Veterinary Sciences, Bahauddin Zakariya University, Multan, 60000 Pakistan; 4 Division of Science and Technology, Department of Botany, University of Education, Lahore, Pakistan; 5 Institute of Microbiology, Faculty of Veterinary Sciences, University of Veterinary and Animal Sciences, Lahore, Pakistan; 6 Department of Biomedical and Diagnostic Sciences, University of Tennessee, Knoxville, USA; 7 MOE Joint International Research Laboratory of Animal Health and Food Safety, College of Veterinary Medicine, Nanjing Agricultural University, Nanjing, 210095 People’s Republic of China; 8 King Abdulaziz City for Science and Technology, Riyadh, 12354 Saudi Arabia; 9 Department of Pharmacology and Toxicology, College of Pharmacy, King Saud University, P.O. Box 2457, Riyadh, 11451 Saudi Arabia

**Keywords:** Pharmaceutics, Parasitology, Drug development, Parasitic infection

## Abstract

Coccidiosis causes huge economic losses worldwide. Current study evaluated the effect of biosynthesized Zinc oxide nanoparticles (ZnONPs) using *Nigella sativa,* on *Eimeria tenella* infected broilers. Scanning electron microscopy showed spherical ZnONPs with 50–100 nm diameter, Fourier transforms infrared spectroscopy revealed the functional groups involved in the reduction of zinc acetate dihydrate to ZnONPs, UV–vis spectroscopy showed a peak at 354 nm, and Zeta potential exhibited stability at − 30 mV. A total of 150, a day-old broiler chicks were divided into 5 equal groups. Control negative: uninfected and untreated; Control positive: Infected and untreated; 3rd, 4th and 5th group were infected orally with 5 × 10^4^ sporulated oocysts of *Eimeria tenella* and treated with 60 mg/kg ZnONPs, 1*% Nigella sativa* seeds and amprolium 125 ppm, respectively. ZnONPs significantly (*p* < 0.05) improved the growth performance in the infected birds and decreased the oocyst shedding and anti-coccidial index. A significant (*p* < 0.05) decrease in the level of aspartate transferase and alanine transferase, whereas, a significantly higher amount of antioxidants like catalase and superoxide dismutase in ZnONPs treated group was observed. Pro-inflammatory cytokines like IL-2 and TNF-α were significantly decreased by ZnONPs (*p* < 0.05). In conclusion, biogenic ZnONPs with *Nigella sativa* might have enhanced anticoccidial, antioxidant, and anti-inflammatory effects with improved growth performance.

## Introduction

The poultry business has become the fastest-growing business in many countries^1^. Parasitic infections are responsible for greater economic losses to the poultry farmers^2^. Coccidiosis is a very fatal protozoal disease characterized by bloody droppings, intestinal inflammation, poor growth performance, increased morbidity and mortality^3^. Coccidiosis affects nutrient digestibility by reducing the size of villi in the small intestine^4^, and alters the expression of genes regulating transport proteins and digestive enzymes of the small intestine^5,6^. In coccidiosis, there is increased production of reactive oxygen species (ROS) and nitrogenous species responsible for lower production of antioxidant enzymes including superoxide dismutase and catalase^7^. Also, liver enzymes like aspartate aminotransferase (AST) and alanine aminotransferase (ALT) increase notably. Financial losses in the diseased birds are linked to poor growth performance and increased death rates as a result of multiple changes in their physiological and metabolic system^8,9^. For control of coccidiosis different anticoccidials are used but resistance develops due to the widespread use of different anticoccidials like amprolium, clazuril, diclazuril, monensin, salinomycin, spiramycin, sulfadimethoxine, and toltrazuril^10^. Currently, natural remedies are adopted as alternatives to treat a variety of ailments^11^.

Anticoccidial drugs in birds have resulted in drug resistance as well as the presence of drug residues in chicken products like meat and eggs, ultimately posing a major public health risk^12^. However, the unavailability of new anticoccidial drugs demands novel anticoccidial alternatives^13^. Nanotechnology methods have currently been incorporated into the veterinary industry, not only for disease diagnosis but also for drug development and prevention of diseases^14^. Zinc is an essential mineral for poultry growth but too much zinc oxide (ZnO) in the feed leads to poor digestion and zinc excretion in the faeces, which harms the environment^15^. A good substitute for traditional zinc sources is the synthesis of zinc oxide nanoparticles (ZnONPs). Nowadays, trace elements such as zinc are manufactured as nanoparticles possessing higher absorption capacity and effectiveness than both natural and anthropogenic sources^16^.

ZnONPs exhibit greater efficacy because of having a large surface area, small particle size, efficient catalysts, more absorption and adsorption in comparison to old zinc sources^17^. Lower dosages of ZnONPs have better therapeutic effect on animals, good chemical activity, participation in oxidative reactions, whereas, these particles are environment friendly due to high bioavailability and good pharmacological activities than traditional zinc oxide^18^. ZnONPs have antimicrobial, antifungal, antiparasitic, anti-diabetic activities and are used as growth promotors, dietary supplements, immune- modulators, and catalysts for phytochemical reactions^19,20^. Furthermore, ZnONPs used in biological sensing, biological labeling, gene transport and targeted drug delivery^21,22^. Previous studies have reported that ZnONPs provided good results in terms of broiler productivity, antioxidant status, and gastrointestinal function^23^. Nanoparticles synthesized from physical and chemical methods have a limited range of size and morphology. These methods are very costly and the chemicals used cause environmental pollution and toxicity in the body. However, biogenic synthesis of nanoparticles with plants is an efficient, environmentally friendly, cost-effective, time saving, and energy saving method^24^. *Nigella sativa* is a medicinal plant of the *Ranunculaceae* family, its chief pharmacologically active ingredient is thymoquinone and it has anti-inflammatory, anti-oxidant, anti-parasitic, and anti-microbial properties^25^. ZnONPs loaded with garlic extract decreased the amount of ALT, AST, and ALP enzymes in rabbits infected with *E. stiedae*^26^. Some investigations have revealed that ZnONPs have antioxidant properties^27,28^. The current study was designed to check the anticoccidial, antioxidant, and anti-inflammatory effects of biosynthesized zinc oxide nanoparticles (ZnONPs) in *Eimeria tenella*-infected boilers.

## Materials and methods

### Preparation of Nigella sativa seed extract

*Nigella sativa* seeds were purchased from the local market of Lahore, Pakistan for green synthesis of Zinc oxide nanoparticles. Seeds were grounded to powder form and then dissolved in distilled water and boiled for 30 min. The mixture was filtered using Whatman filter paper No.1, and the extract was stored at 4 °C^29^.

### Green synthesis of zinc oxide nanoparticles

**Figure 1:**
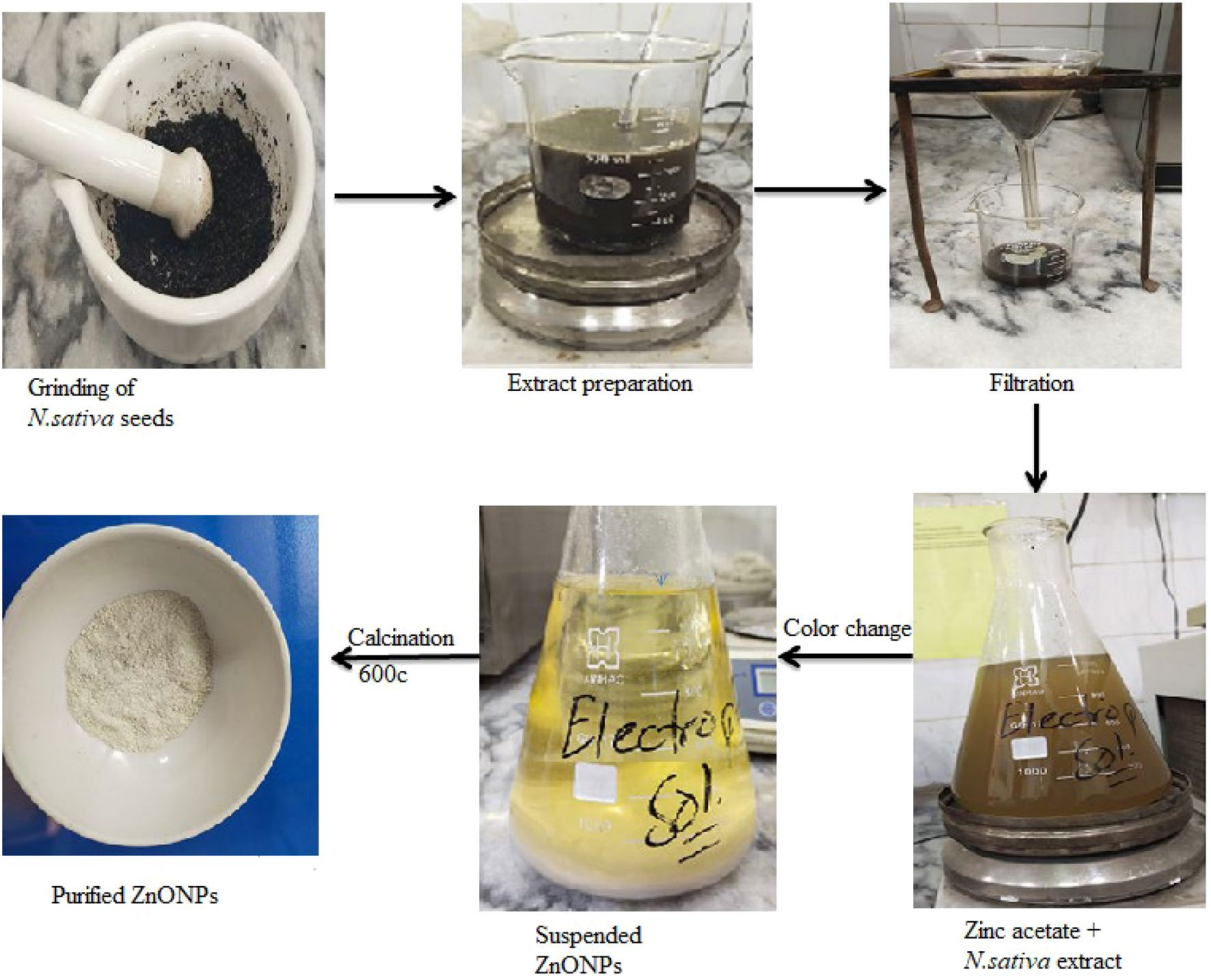
Green synthesis of zinc oxide nanoparticles by using *Nigella sativa* as a reducing agent.

**Figure 2:**
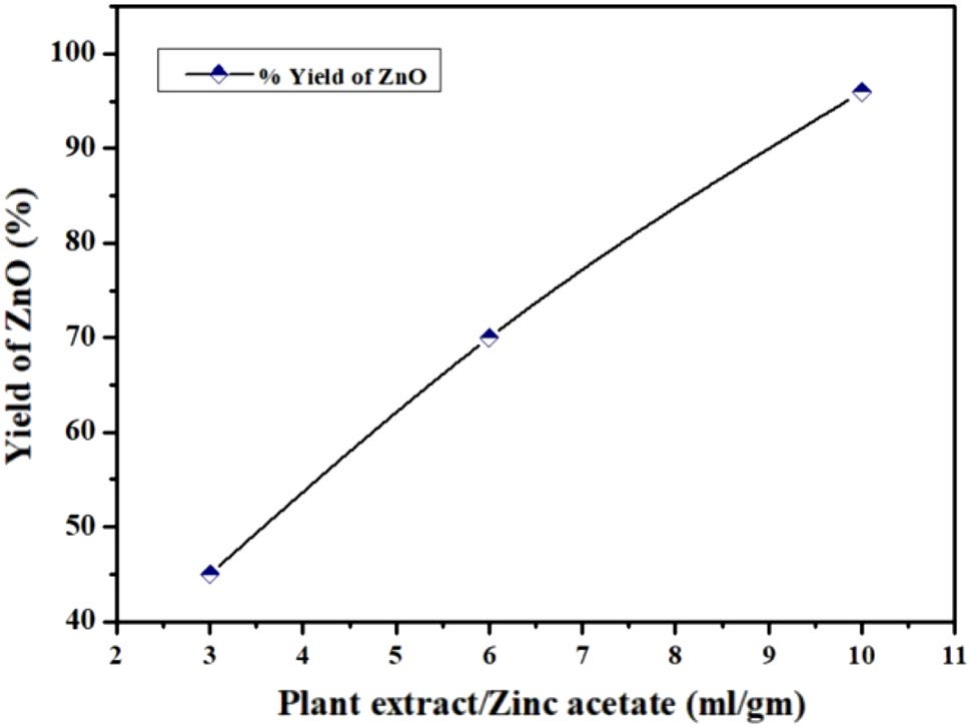
Effect of the plant extract to Zinc acetate ration (v/w) on yield (%) of Biosynthesized ZnONPs.

Zinc acetate dihydrate salt (0.25 M) solution was prepared by adding 22.92 g zinc acetate dihydrate salt to 500 ml of distilled water in a flask. Further, 10 ml of *Nigella sativa* seed extract added dropwise into the zinc acetate solution under continuous stirring at 60 ℃ for 2 h on a magnetic stirrer. While stirring, NaOH was added dropwise in the above solution to maintain the pH at 12. Plant extract acted as a reducing agent. The formation of light yellow color suspended particles and visible color change of the solution from brown to yellow indicated the formation of zinc oxide nanoparticles. This mixture was then centrifuged at 6000 × g for 15 min and supernatant was discarded to collect the pellet in a Petri plate. Afterwards, it was placed overnight in a hot air oven for drying at 60 ℃. Calcination of yellow-colored ZnONPs was done in a muffle furnace at 400 ℃ for 4 h to remove impurities (Figs. 1, 2)^30^.

Influence of the plant extract on production of ZnONPs was investigated by varying the ratio of plant extract to salt (v/w) and yield of prepared ZnONPs was calculated by following formula

$$ {\text{Yield }}\;\% \;{\text{age }} = {\text{ Experimental}}\;{\text{ ZnO}}\;{\text{ weight}}/{\text{Theoretical}}\;{\text{ ZnO}}\;{\text{ weight }} \times {1}00 $$

### Characterization of zinc oxide nanoparticles

The optical properties of Zinc oxide nanoparticles (ZnONPs) were analyzed by using a UV- spectrophotometer. Firstly, water was run as a blank, and then the ZnONPs sample was set in a spectrophotometer. UV light passed at 354 nm and confirmed the formation of ZnONPs^31^. Scanning Electron Microscopy was used to identify the morphology of ZnONPs. The sample was observed at 20kv with a frequency of 2838cps. For determination of shape and size, ZnONPs was analyzed at different resolution and magnification power. Fourier transform infrared spectroscopy (FTIR) analysis was performed to know the functional groups involved in the synthesis of ZnONPs. The solution was dried at 75˚C and characterization was done at a wavenumber ranging from 4000 to 400 cm^−1^^29^. Stability of green synthesized ZnONPs were analyzed by Zeta potential^32^. To determine the purity and structural characteristics of ZnONPs, diffraction intensities was recorded at 30 kV and 10 mA current with the range 2*θ* from − 3 to 160°by using X-ray diffractometer (Bruker D2 Phaser).

### Preparation of Eimeria tenella infection

Feces from *E.tenella* infected bird’s ceca were collected and examined for the presence of oocysts by sedimentation technique. By floatation technique, *E.tenella* oocysts were collected from the supernatant and washed with tap water by centrifugation at 1500 rpm for 10 min. For sporulation, washed oocysts were incubated at room temperature in 2.5% potassium dichromate for 72 h. Sporulated oocysts were washed with PBS by centrifugation at 1500rom for 10 min. Beading was performed by vortexing the sporulated oocysts with 0.5 mm sterilized glass beads^33^.

### Ethical approval

The experiments were performed in accordance with comprehensive animal welfare guide (FASS, 2010) and approved by the Ethical Review Committee (ERC) of the University of Veterinary and Animal Sciences (UVAS), Lahore, Pakistan (permit No. DR/ 357).

## Experimental design

The experiments were performed in accordance with comprehensive animal welfare guide (FASS, 2010) and approved by the Ethical Review Committee (ERC) of the University of Veterinary and Animal Sciences (UVAS), Lahore, Pakistan (permit No. DR/ 357). This study also followed the ARRIVE guidelines. A total of 150, day-old broiler chicks were purchased from a nearby hatchery and kept under standard conditions. Birds were divided into five equal groups. On the 28th day except for group 1, all groups were infected orally through a stomach tube, with 5 × 10^4^ sporulated oocysts of *Eimeria tenella.* Group 1 was control negative, which was kept un-infected and un-medicated. Group 2 was control positive, infected, and un-medicated. Group 3 was fed with 60 mg/kg ZnONPs orally through a stomach tube. Group 4 was fed with 1% *Nigella sativa* crushed seeds mixed in feed. Group 5 was given amprolium at a dose rate of 125 ppm orally. Treatment was given from 28 to 35th day of trial.

### Anticoccidial activity analysis

Anticoccidial activity of ZnONPs was analyzed by recording the body weight gain (BWG), FCR, OPG, mortality rate, lesion scores, and anticoccidial index of the experimentally infected birds.

### Determination of weight gain and feed conversion ratio (FCR)

Growth performance, body weight, and feed intake of the birds were recorded on weekly basis^34^.

### Fecal examination

Fecal samples of birds were collected daily, post challenge till 35th day. McMaster oocyst counting technique was used for quantitative examination of feces, while qualitative examination of feces was done by using the direct smear and flotation techniques^35^.

### Mortality rate and lesion score

Birds were observed daily for mortality. At the end of the trial, birds from each group were slaughtered and examined for cecal lesions, and scored on the basisof severity of lesions. 0 score for –ve group and 4 for severely affected and dead birds^36^.

### Anticoccidial index (ACI)

Anticoccidial index of each group was calculated by the formula: (Relative ratio of the body weight gain + survival rate)–(lesion index + oocyst value). Anti-coccidial index greater than 180 is perceived to have a very effective anticoccidial action. ACI in 160–180 range indicated marked anticoccidial action and ACI less than 120 indicated no anticoccidial activity^37^.

$$ {\text{Oocyst}}\;{\text{value}}\; \, \% \;{\text{calculated}}\;{\text{as}} = {\text{OPG}}\;{\text{of}}\;{\text{specific}}\;{\text{group}}/{\text{OPG}}\;{\text{of}}\;{\text{control}}\; + {\text{ve}}\;{\text{ group}} \times {1}00 $$

$$ {\text{Survival}}\;{\text{rate}}\;{\text{calculated}}\;{\text{as}} = {\text{No}}.\;{\text{of}}\;{\text{ chickens}}\;{\text{survived}}/{\text{Original}}\;{\text{No}}.\;{\text{of}}\;{\text{chickens}} \times {1}00 $$

$$ {\text{Relative}}\;{\text{BWG}}\;{\text{ratio}}\;{\text{calculated}}\;{\text{as}} = {\text{Avg}}.\;{\text{BWG}}\;{\text{in}}\;{\text{specific}}\;{\text{group}}/{\text{Avg}}.\;{\text{BWG}}\;{\text{in}}\;{\text{control}}\;{-}{\text{ve}}\;{\text{group}} \times {1}00 $$

### Blood sampling and serum biochemical analysis

Blood samples (2 ml) were collected on 35th day from the wing vein of all the birds in plain tubes, allowed to clot and then centrifuged at 4000 rpm for 10 min. The obtained serum was stored at − 20 °C for biochemical analysis^38^. Serum biochemical profile including aspartate transferase (AST), and alanine transferase (ALT) was evaluated by using a Diasys kit following the manufacturer’s guidelines^39^.

### Determination of antioxidant enzymes activity

Antioxidants like catalase (CAT) and superoxide dismutase (SOD) activities were evaluated by using Fine test Elisa kits following guidelines given by the manufacturer^40^.

### Determination of serum inflammatory cytokines

The determination of tumor necrosis factor-alpha (TNF-α) was evaluated by using the BTlab ELISA kit and level of Interleukins-2 (IL-2) was determined by using a Fine test ELISA kit following the manufacturer’s guidelines^41^.

### Statistical analysis

The data were analyzed by using one-way ANOVA using SPSS version 20.0 and difference between mean was determined using Tukey’s test at (*p* < 0.05) level.

## Results

### Ultra violet analysis of zinc oxide nanoparticles

**Figure3:**
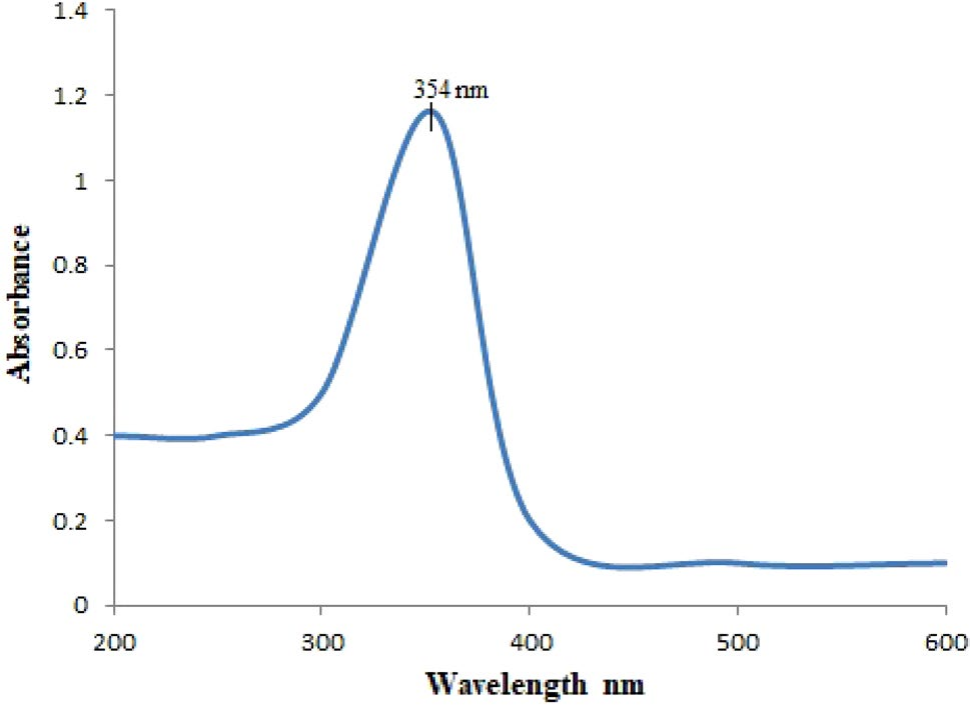
Ultra violet visible spectrum of *Nigella sativa* mediated zinc oxide nanoparticles.

Ultraviolet spectroscopy confirmed the synthesis of ZnONPs and showed the optical activity and stability of nanoparticles. After the formation of yellow suspended particles, this solution was run in an ultraviolet spectrophotometer. The light passed and spectra observed at 354 nm indicated ZnONPs as shown in (Fig. 3).

### Scanning electron microscopy

**Figure 4:**
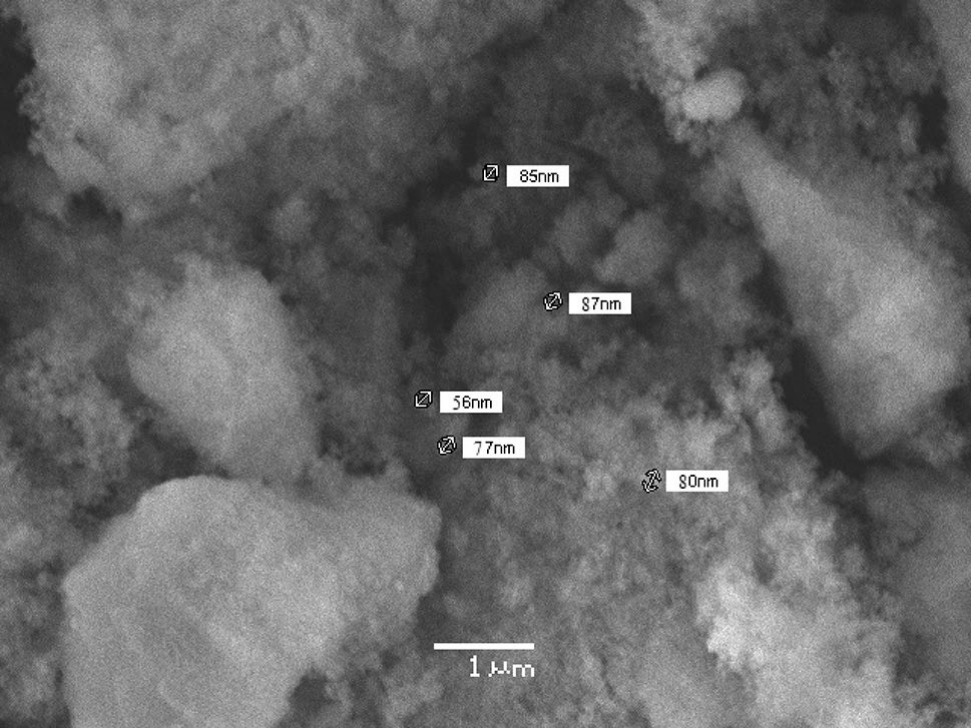
Scanning electron microscopy image of zinc oxide nanoparticles showing diameter at 1 µm resolution.

Scanning electron microscopy images showed spherical-shaped nanoparticles of average size ranging from 50–100 nm (Fig. 4).

### X-ray diffraction analysis

**Figure 5:**
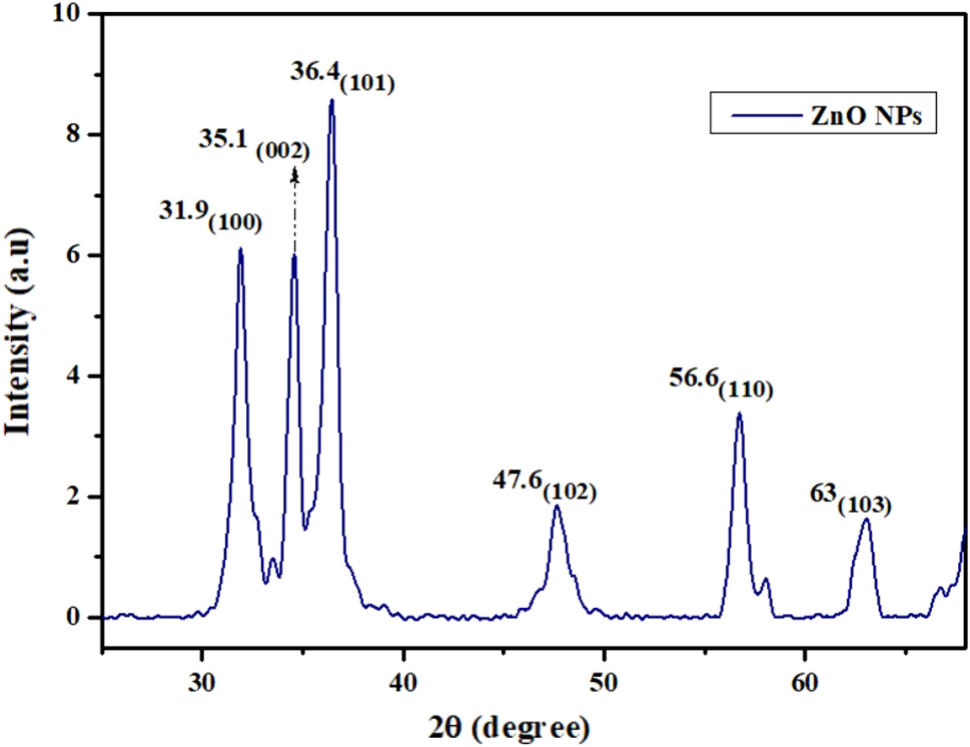
X-ray Diffraction analysis of *Nigella sativa* mediated ZnONPs.

XRD peaks showed 2*θ* values at 31.9, 35.1, 36.4, 47.6, 56.6 and 63 corresponding to the (100), (002), (101), (102), (110) and (103) planes showing hexagonal wurtzite crystalline ZnONPs using JCPDS (Fig. 5).

### Fourier transform infra-red spectroscopy

**Figure 6:**
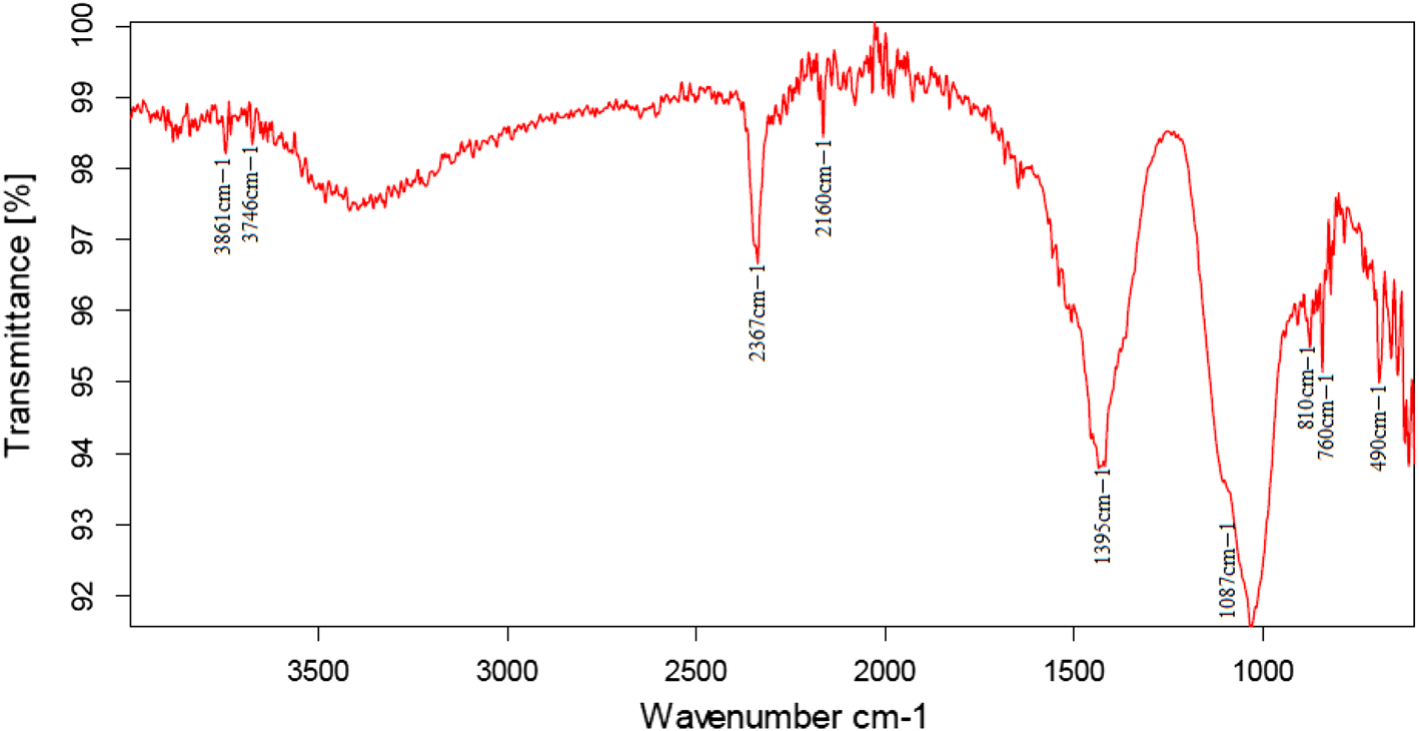
Fourier transform infra-red spectra of zinc oxide nanoparticles biosynthesized by *Nigella sativa* as reducing agent.

Fourier transform infrared spectrum of ZnONPs showed several peaks at 3861, 3746, 2367, 1395, 1087, 850, 760, and 490 cm^−1^ (Fig. 6).

### Zeta potential

**Figure 7:**
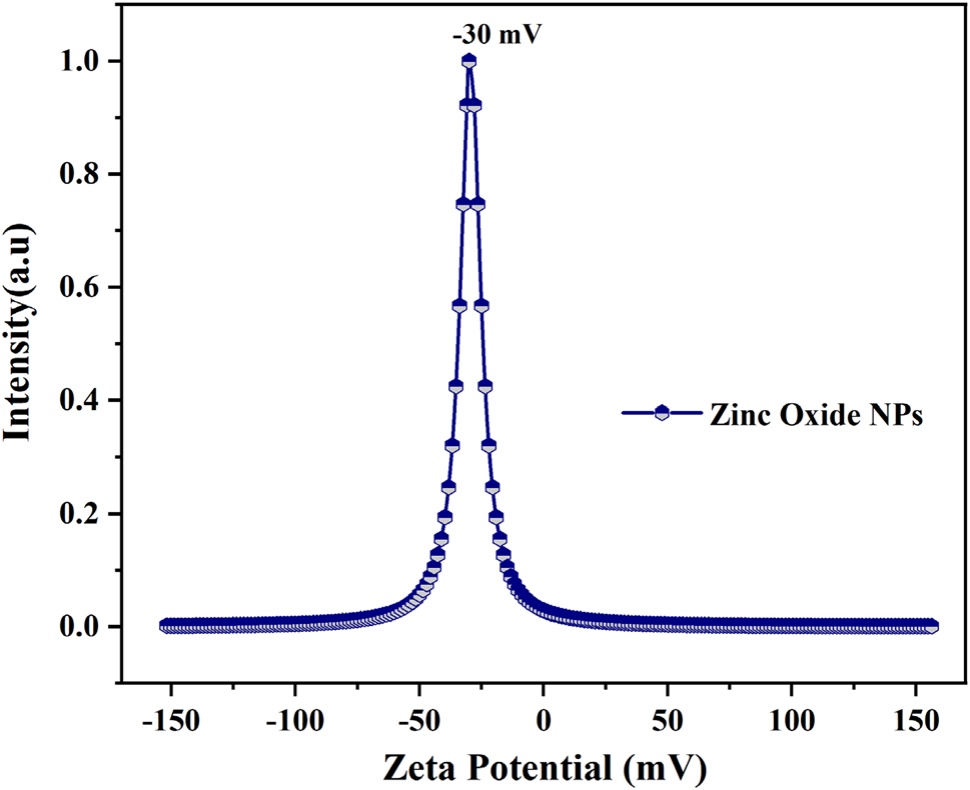
Stability of *Nigella sativa* mediated zinc oxide nanoparticles.

Results of zeta potential at − 30 mV indicated the colloidal stability of green synthesized ZNONPs (Fig. 7).

### Growth performance

An increase in body weight was noted in birds of all groups and there was no significant difference (*p* > 0.05) among different groups till the onset of clinical signs of the coccidiosis. Growth performance decreased significantly (*p* < 0.05) due to *Eimeria* infection. Treatment with ZnONPs and *Nigella sativa* significantly (*p* > 0.05) enhanced the growth performance and the results were comparable to that of the amprolium-treated group (Table 1).

**Table 1:** Growth performance of the broiler birds challenged with *Eimeria tenella* and given different treatments. (Mean ± S.D.)

BW on day (g)	Control –ve	Control + ve	ZnONPS	NS seeds	Amprolium	SEM	*p*-value
0 day	39.3 ± 0.3^a^	39.8 ± 0.3^a^	39.5 ± 0.5^a^	39.9 ± 0.6^a^	39.9 ± 0.6^a^	.110	.298
21^st^ day	925.8 ± 13.2^a^	924.0 ± 12.9^a^	926.0 ± 12.9^a^	926.4 ± 13.5^a^	924.8 ± 14.5^a^	2.461	.999
35^th^ day	1902.0 ± 25.8^a^	1430.0 ± 21.5^b^	1851.6 ± 21.5^c^	1844.8 ± 22.5^c^	1840.6 ± 29.1^c^	35.655	.000
BWG on day (g)							
0-21 days	886.7 ± 13.6^a^	884.4 ± 14.5^a^	886.7 ± 13.6^a^	886.7 ± 13.6^a^	884.4 ± 14.5^a^	2.573	.997
21-35 days	976.2 ± 20.1^a^	506.6 ± 27.3^b^	925.7 ± 24.0^c^	918.8 ± 15.7^c^	916.56 ± 13.9^c^	35.41	.000
0-35 days	1862.5 ± 23.8^a^	1390.6 ± 21.3^b^	1812.2 ± 18.8^c^	1804.9 ± 20.6^c^	1800.5 ± 24.3^c^	35.58	.000
Feed intake during period (g)							
0-21 days	1258.6 ± 13.1^a^	1233.4 ± 19.6^a^	1243.8 ± 16.5^a^	1245.2 ± 18.0^a^	1235.6 ± 17.4^a^	3.611	.192
21-35 days	1546 ± 9.5^a^	1371 ± 8.4^b^	1574 ± 4.4^c^	1586 ± 9.8^c^	1587 ± 4.8^c^	16.9	.000
0-35 days	2900 ± 7.9^a^	2700 ± 7.9^b^	2992 ± 8.1^c^	3020 ± 7.2^d^	3011 ± 11.4^d^	24.5	.000
Feed conversion ratio during period feed (g)/weight gain (g)							
0-21 day	1.41 ± .05^a^	1.39 ± .02^a^	1.40 ± .02^a^	1.40 ± .02^a^	1.39 ± .02^a^	.006	.631
21-35 day	1.58 ± .02^a^	2.70 ± .15^b^	1.70 ± .03^ac^	1.72 ± .03^c^	1.73 ± .03^c^	.084	.000
0-35 day	1.55 ± .02^a^	1.94 ± .01^b^	1.65 ± .02^c^	1.67 ± .02^c^	1.67 ± .03^c^	.026	.000

Values are Mean ± S.D, Control –ve = Un-infected and un-treated, Control + ve = Infected and un-treated, ZnONPs = Infected and treated with 60 mg/kg ZnONPs orally, NS seeds = Infected and treated with 1% Nigella sativa crushed seeds, Amprolium = Infected and treated with amprolium. BW: body weight; BWG: body weight gain; FI: feed intake; FCR: feed conversion ratio (FI/BWG); SEM: standard error of mean. ^a,b, c,d^ mean within a row with different superscripts are significantly different at (p < 0.05).

### Oocysts shedding and lesion scores

On the 4th day post-infection, there was frequent bloody diarrhea in the infected groups and generalized weakness was present in the birds. No. of oocysts per gram (OPG) of feces was counted by McMaster’s chamber on, 32th, 33th, 34th, and 35th days. On the 4th day post-infection, a large number of oocysts was shedding in the feces and maximum OPG was found in the control positive group, which was significantly higher (*p* < 0.05) than in other groups. The non-infected group was free from any signs and oocyst shedding. In treatment groups, OPG of ZnONPs groups was lower than in NS seeds and amprolium treated groups. Lesion score was significantly higher in control + ve group indicating the cecal damage. Lesion scores in ZnONPs were significantly low (*p* < 0.05) and comparable to that of amprolium treated group (Table 2).

**Table 2:** Oocyst per gram (× 10^4^) and caecum lesion scores of the broiler birds challenged with *Eimeria tenella* and treated with different treatments at 28–35 days of age. (Mean ± S.D).

Groups	OPG				Lesion scores
	32nd day	33th day	34th day	35th day	
Control -ve	0.0 ± 0.0^a^	0.0 ± 0.0^a^	0.0 ± 0.0^a^	0.0 ± 0.0^a^	0.0 ± 0.0^a^
Control + ve	9.9 ± 0.69^b^	8.5 ± 0.68^b^	5.9 ± 0.63^b^	3.4 ± 0.51^b^	2.6 ± 0.46^b^
ZnONPS	5.0 ± 0.97^c^	2.5 ± 0.60^c^	1.1 ± 0.15^c^	0.11 ± 0.008^c^	0.7 ± 0.19^c^
NS seeds	6.7 ± 0.48^d^	3.9 ± 0.86^d^	2.0 ± 0.23^d^	0.54 ± 0.008^c^	1.4 ± 0.33^d^
Amprolium	5.2 ± 0.85^c^	2.2 ± 0.79^c^	1.3 ± 0.39^c^	0.24 ± .033^c^	0.6 ± 0.19
SEM	0.704	0.592	0.420	0.266	0.186
*p*-value	0.000	0.000	0.000	0.000	0.000

Values are Mean ± S.D. Control –ve = Un-infected and un-treated, Control + ve = Infected and un-treated, ZnONPs = Infected and treated with 60 mg/kg ZnONPs orally, NS seeds = Infected and treated with 1% Nigella sativa crushed seeds, Amprolium = Infected and treated with amprolium; SEM: standard error of mean. ^a,b, c,d^ mean within a column with different superscripts are significantly different at (p < 0.05).

### Anticoccidial index (ACI)

**Figure 8:**
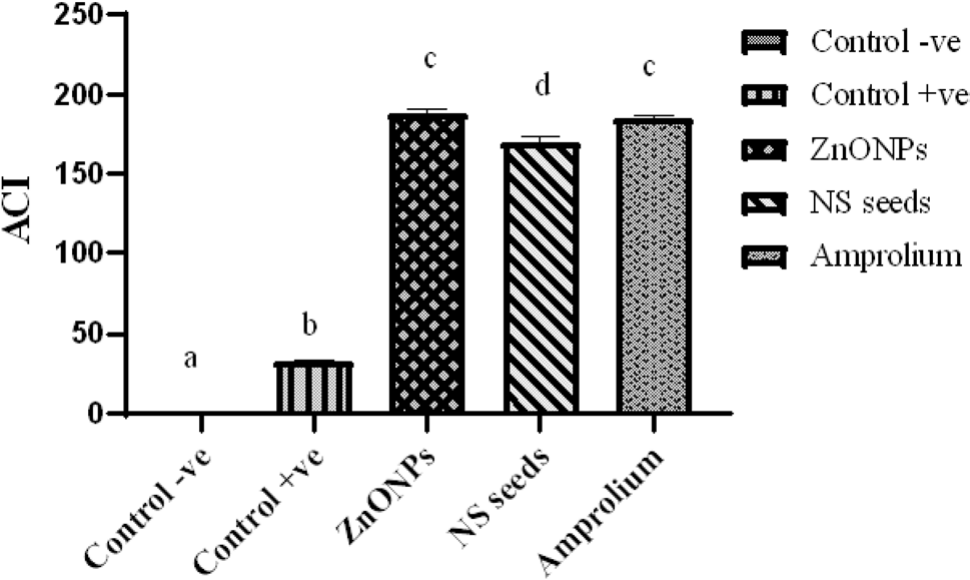
Anti-coccidial Index of the broilers challenged with *Eimeria tenella* and given different treatments. Bars with different superscripts are significantly different at (*p* < 0.05).

There was 16% mortality in the control positive group and no mortality in the treated and control negative group. Lesion index was calculated by multiplying lesion score by 10. ACI of the control positive group was < 120 indicating no anticoccidial effect. ZnONPs treated group showed ACI > 180 comparable to that of amprolium treated group (*p* > 0.05) (Fig. 8).

### Serum biochemical analysis

The current study observed an increased level of ALT and AST in the serum due to tissue damage 7 days post-infection (Fig. 8). Amount of ALT and AST showed a significant (*p* < 0.05) increase in an infected group as compared to the non-infected group. Group treated with ZnONPs has shown a significant effect (*p* < 0.05) on the amount of ALT and AST comparable to amprolium treated group.

### Antioxidant enzyme activities

The current study observed a significant (*p* < 0.05) decrease in the amount of SOD and CAT in control positive group. The level of SOD and CAT was significantly increased (*p* < 0.05) in infected but treated groups. ZnONPs showed a significant effect (*p* < 0.05) as compared to other treatment groups (Fig. 8).

### Serum inflammatory cytokines

**Figure 9:**
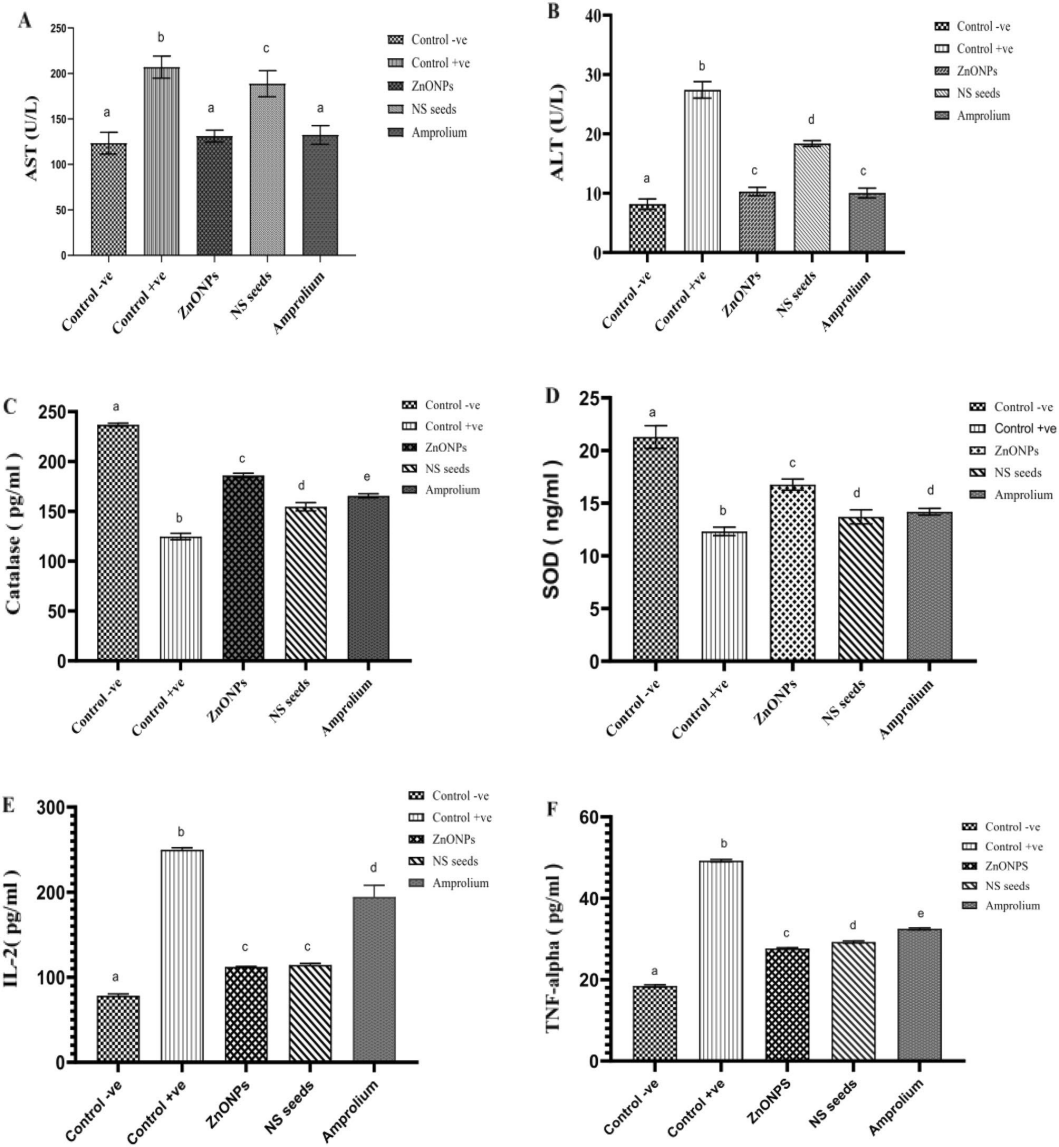
Serum biochemical profile, Antioxidant status, and level of inflammatory cytokines of the broilers challenged with *Eimeria tenella* and given different treatments (**A**) Aspartate transferase, (**B**) Alanine transferase, (**C**) Catalase, (**D**) Superoxide dismutase, (**E**) Interleukins-2, (**F**) Tumor necrosis factor-α.

The amount of IL-2 and TNF-α was significantly higher (*p* < 0.05) in the infected group than control negative and other treated groups. ZnONPs and NS seed groups showed a significant decline in the level of IL-2 and TNF-α. The level of IL-2 and TNF-α in amprolium group was significantly lower (*p* < 0.05) than that of control positive group but higher than other groups (Fig. 9).

## Discussion

Poultry coccidiosis is a very harmful disease that damages the gastrointestinal tract of the birds causing huge economic losses. Prevailing resistance against available anticoccidials provokes the use of alternative treatments for coccidiosis like natural products^42^. Moreover, the drugs used against coccidiosis like toltrazuril cause the problem of drug residues in poultry products i.e. meat and eggs^43^. That’s why nanoparticles and herbal plants are being used as a new and effective remedy against coccidiosis with maximum efficacy and no residue problem^44^.

In this study, anticoccidial effect of green synthesized ZnONPs was evaluated as compared to traditional zinc. During green synthesis, a color change from light brown to yellow was observed indicating the reduction of zinc^45^. Bioactive components like quinones, alkaloids, phenolics and flavonoids present in *Nigella sativa* extract reduced the zinc acetate salt and control the size of ZnONPs. Carboxylic and amino groups of these bioactive compounds binds to the surface of zinc (Zn^+2^) and amides from capping proteins may act as a stablizer^46^. Plant extract concentration is critical in the production of ZnO NPs. Hence, 3 ml/gm and 6 ml/gm don’t have sufficient bioactive components that can reduce Zinc acetate and lead to low yield of NPs. Whereas, 10 ml/gm has given highest yield of ZnONPs^47^ (Fig. 2). Confirmation of synthesis of ZnONPs was done by Uv- analysis. In Uv-spectrum, a clear absorption peak was formed at 354 nm, indicating the presence of ZnONPs. In another study by^48^, ZnONPs were synthesized by using *Cassia fistula* and *Melia azedarach* which revealed absorption peak at 320 nm and 324 nm, respectively^49^ reported that ZnONPs synthesized by leaf extract of *Piper betle* showed absorption peaks at 358, 368, and 378 nm. This low absorbance is due to presence of complex biomolecules in the plants^50^.

Scanning Electron Microscopy images showed more or less spherical NPs of 50–100 nm size range^51^ reported that the diameter of ZnONPs synthesized by *Mentha Spicata* extract was 11–80 nm. The difference in the size of the NPs is because of the difference in temperature conditions provided during the synthesis of NPs. If a high temperature is provided to the reaction mixture, small-sized NPs will be synthesized^52,53^. The volume of the plant extract also plays a role in controlling the size and shape of NPs. Using a large volume of extract during synthesis will reduce the size of NPs^54,55^. FTIR spectrum showed a peak at 3861 cm^−1^ and 3746 cm^−1^ representing the stretching vibration of hydroxyl OH-bond from phenolic compounds present in the plant^29^. The peak at 2367 cm^−1^ indicated N–H bond of the primary and secondary amides. The band at 1395 cm^−1^ indicated C=O stretching of amides and C=C stretching of alkenes^56^. The band at 1087 cm^−1^ corresponded with stretching of C-O bond^57,58^. The peaks at 850 cm^−1^ and 760 cm^−1^ indicated C–N stretch of the amine group^59^. The absorption peak at 490 cm^−1^ showed the ZnO bond as reported previously^60^. The presence of alkenes is confirmed by FTIR spectra that have a role in antioxidant activity^61^. Hydroxyl bonds confirmed the presence of flavonoids, having antioxidant and anti-inflammatory properties^62^ and C=O groups indicateed the presence of thymoquinone responsible for anti-coccidial activity^63^. Zeta potential of − 30 mV showed good stability of green synthesized ZnONPs. Negative surface charge is due to the strong binding affinity of extract chemicals for the NPs which increased their stability and reduced their tendency to aggregate^64^. Distinct narrow peaks of XRD showed that ZnONPs are free from impurities and have hexagonal wurtzite crystals^47^.

In this study, a significant decrease in growth performance was observed in an infected group similar to the studies reported by^65^. The reduction in the growth performance may occur due to intestinal damage and decreased length of the villi which may have resulted in poor nutrient absorption^66^. Infected but treated groups, with ZnONPs, NS seeds, and amprolium respectively, showed improvement in growth performance because of decreased intestinal damage^36^ reported that the supplementation of the *Nigella sativa* reduced the adverse effects of *Eimeria* and increased the growth performance. ZnONPs are better than the traditional Zn sources in improving feed utilization and growth because Zn is involved in many enzymatic reactions of metabolism^67^. ZnONPs promote growth because they increase the intestinal villi length and crypt depth and hence resulting in increased nutrient absorption^68^.

The results of this study showed that there was severe bloody diarrhea in the control positive group and a maximum number of oocysts shedding in the feces. Bloody diarrhea occurred due to penetration of spores inside the cecal epithelium and spores of protozoans complete their developmental stages inside the epithelium till the schizogony stage, ultimately damaging the intestinal blood vessels and causing hemorrhages^69,70^. Current study revealed that ZnONPs significantly decreased the number of *E. tenella* oocysts in the feces, caecum lesion score and anticoccidial index at a dose of 60 mg/kg similar to^71^. This may be due to the reason that ZnONPs diminished the growth and development of protozoa inside the intestinal cells before the inactive oocysts were formed and shed^44^. Furthermore, NS seeds given in diet also decreased the OPG in *E.tenella-*infected birds^36^.

Current study showed a significant increase level of AST and ALT in the serum of infected birds. Similar results were reported by^10^ and they proposed that this rise in enzyme activity occurs due to cecal cell wall damage, inflammation, and heavy blood loss. In this study, ZnONPs and NS seeds caused a reduction in the level of these enzymes. It is thought that quinone component present in NS seeds may have a role in reducing cecal lesions^72^. Our results are in line with the study of^36^ which showed that the treatment with NS seeds reduced the level of these enzymes. In contrast^73^, reported that there was no significant change in the AST and ALT levels in infected and treated groups. Moreover^74^, stated that the level of ALT was enhanced after *E. tenella* infection, but there was no effect of infection on level of AST. This contradiction in results may occur because of variations in the degree of intestinal inflammation, intestinal damage and hemorrhages^75^.

Results of antioxidant activity showed a significant decrease in the amount of SOD and CAT, indicating oxidative damage because of *Eimeria* infection^76^. While the level of SOD and CAT was high in treatment groups similar to the findings of^77^ who stated that *Nigella sativa* significantly increased the level of SOD and CAT. In our study, the antioxidant activity of ZnONPs was higher than the other treated groups similar to the results of^78,79^ who stated that administering ZnONPs in the broiler diet significantly increases both SOD and CAT activity in serum. However, in contrast to our study, (El Katcha et al. 2017) reported that there was no significant change in antioxidant activity upon feeding 15–60 ppm nano-Zinc to the broilers. Moreover, studies have reported that 60 mg/kg ZnONPs have good Cu–Zn-SOD activity. The reason for the marked antioxidant property of ZnONPs is that Zn is an important constituent of SOD and this enzyme reduces oxidative stress by efficiently scavenging the superoxide free radicals^80^. Another mechanism behind the antioxidant activity of ZnONPs is the competition of zinc with iron and copper for attachment on the binding site present on cell membranes and that’s why Zn decreases free radical production^81^. Moreover, it is also proposed that Zn promotes the production of metallothionein and this protein plays an important role in the detoxification of free radicals^82^ and also activates the antioxidant proteins and enzymes like glutathione peroxidase (GPx) and CAT^83^.

This study indicated a significantly high level of pro-inflammatory cytokines in broilers of the control positive group^84^ showed that in intestinal inflammation, pro-inflammatory cytokines intensely increased because these are attracted to the site of inflammation. Treatment with biogenic ZnONPs diminished the pro-inflammatory cytokines (IL-2 and TNF-α). Our results are in agreement with^85^, who reported that ZnONPs decreased the mRNA expression of IL-2 and TNF-α in serum. The anti-inflammatory property of Zn may be due to Nrf2/HO-1 signal pathway^86,87^. Substances that activates the Nrf2 pathway, decrease the overproduction of pro-inflammatory cytokines like IL-2 and TNF-α, and enhances immunoglobulin production^88,89^^,^^90,91^ also stated that an activated Nrf2/HO-1pathway; stops the TLR4-mediated inflammatory responses. It is shown that ZnONPs have anticoccidial property and improved growth performance.

## Conclusion

Biogenic ZnONPs supplementation to *Eimeria tenella* infected broilers showed anticoccidial activity by decreasing the oocysts shedding and improve the growth performance. Moreover, ZnONPs decreased the tissue damage which was indicated by decrease in the level of serum enzymes like alanine transferase and aspartate transferase. ZnONPs showed antioxidant and anti-inflammatory activity by significantly increasing the activity of superoxide dismutase alongwith catalase and decreasing the amount of pro-inflammatory cytokines interleukins-2 as well as tumor necrosis factor-α. So, ZnONPs can be used as an alternative to treat coccidiosis. But the exact mechanism of its anti-coccidial action is still unknown so further research is needed to understand its mechanism of action alongwith comparison between different plant mediated synthesis of ZnONPs.

## Data Availability

All data generated or analyzed during this study are included in this manuscript.
